# Uterine fibroids — Causes, impact, treatment, and lens to the African perspective

**DOI:** 10.3389/fphar.2022.1045783

**Published:** 2023-01-10

**Authors:** Narvella Sefah, Sithembinkosi Ndebele, Lillian Prince, Elizabeth Korasare, Michael Agbleke, Annabella Nkansah, Humphrey Thompson, Ayman Al-Hendy, Andrews Akwasi Agbleke

**Affiliations:** ^1^ University of Chicago, Chicago, United States; ^2^ Fibroid Foundation Africa, Accra, Ghana; ^3^ Sena Institute of Technology, Penyi, Ghana

**Keywords:** fibroid, Africa, causes and treatment, perspective, types and classification, race, lifestyle

## Abstract

Leiomyomas, or uterine fibroids as they are commonly known, are mostly seen in women of reproductive age. However, they can go undetected in most women, and approximately 25% of women show clinical symptoms. Although fibroids are a global burden impacting 80% of premenopausal women, they are more prevalent among Black women than among women of other races. Based on clinical diagnosis, the estimated cumulative incidence of fibroids in women ≤50 years old is significantly higher for black (>80%) *versus* white women (∼70%). The cause of leiomyomas is not clearly known, but studies have shown evidence of factors that drive the development or exacerbation of the disease. Evidence has linked risk factors such as lifestyle, age, environment, family history of uterine fibroids, and vitamin D deficiencies to an increased risk of uterine fibroids, which impact women of African descent at higher rates. Treatments may be invasive, such as hysterectomy and myomectomy, or non-invasive, such as hormonal or non-hormonal therapies. These treatments are costly and tend to burden women who have the disease. Sub-Saharan Africa is known to have the largest population of black women, yet the majority of uterine fibroid studies do not include populations from the continent. Furthermore, the prevalence of the disease on the continent is not well determined. To effectively treat the disease, its drivers need to be understood, especially with regard to racial preferences. This paper aims to review the existing literature and build a case for conducting future research on African women.

## Introduction

Uterine fibroids, also known as leiomyomas, are tumors made of smooth muscle and connective tissue from the myometrium or muscular outer layer of the uterus (Nowak, 1993; Fleischer et al., 2008; Suo et al., 2009; Bulun, 2013; Ke et al., 2013). They can be found in premenopausal women and are observed to regress post-menopause (Levy, 2008; Giannubilo et al., 2015; Sarkodie et al., 2016; Ghosh et al., 2018; Giuliani et al., 2020; Ulin et al., 2020; Ali et al., 2021). Uterine fibroids are common in over 70% of women by the onset of menopause and are clinically apparent in 25% of women of reproductive age (Stewart et al., 2017). These tumors are benign neoplasms and are not predicted to lead to cancer (Bulun, 2013; Orellana et al., 2021).

Fibroids can form in various locations around the uterus and can take different forms (Bulun, 2013; Zepiridis et al., 2016). Approximately 20%–50% of women with fibroids show symptoms of heavy menstrual bleeding, which can lead to anemia, bladder dysfunction, and pregnancy complications (Khan et al., 2014; Stewart et al., 2017; Marsh et al., 2018). Notably, most fibroids go undetected by women and may be small and asymptomatic (Laughlin et al., 2011; Zimmerman et al., 2012; Sheng et al., 2020). Although extensive research has been done, it is still inconclusive as to what causes some fibroids to be asymptomatic and others symptomatic (Divakar, 2008; Marsh et al., 2013; Giuliani et al., 2020). It is hypothesized that the size and location of the fibroid may play a role. Fibroids up to the size of a watermelon have been recorded, while some are as small as a tiny stone (Peddada et al., 2008; Fasubaa et al., 2018; Maanongun et al., 2021). Fibroids can be singular tumors or, less commonly, a cluster and are not limited in size (Hodge et al., 2008; Bulun, 2013).

## Location

**FIGURE 1:**
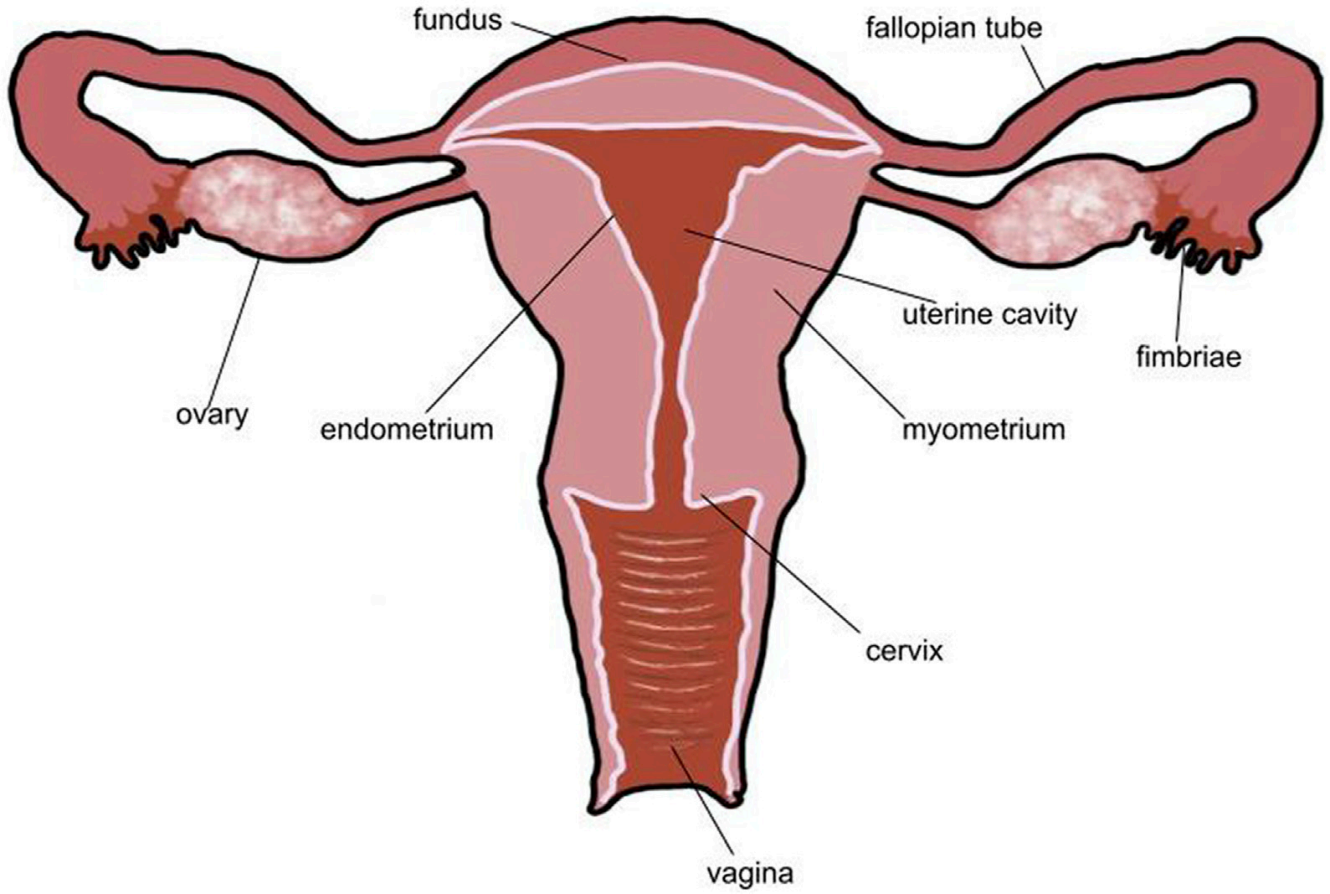
Typical healthy uterus. In a healthy uterus, there are no lesions. The endometrium is a thin layer that surrounds the uterine cavity and myometrium. Both fallopian tubes and ovaries are present. The uterine cavity is empty. No part of the uterus is distended or disformed.

**FIGURE 2:**
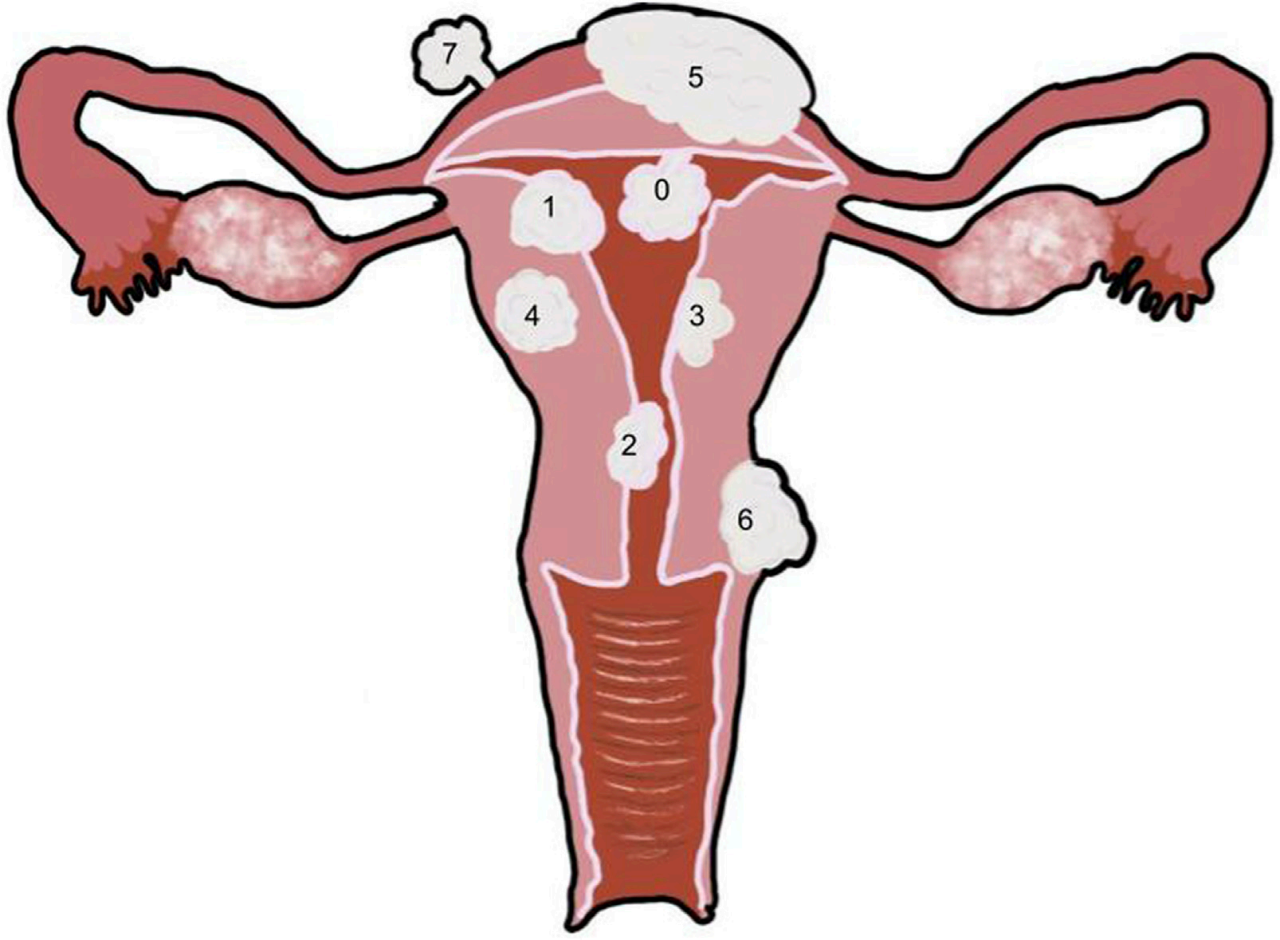
Uterus with multiple fibroid types 0: Pedunculated submucosal, 100% of the fibroid is in the uterine cavity. 1: Submucosal, greater than 50% of the fibroid is within the myometrium and the other portion is distorting the endometrium and uterine cavity. 2: Submucosal, less than 50% of the fibroid is within the myometrium and the majority is distorting the endometrium and uterine cavity. 3: Intramural, the fibroid is within the myometrium but touches the endometrium, and it does not distort the uterine cavity. 4: Intramural, the fibroid is completely within the myometrium. 5: Intramural, the fibroid is predominantly within the myometrium with less than 50% extending outside of the myometrium. 6: Subserosal, greater than 50% of the fibroid is located outside of the myometrium. 7: Pedunculated subserosal, 100% of the fibroid is outside of the myometrium. See Table 1 for classification details.

**FIGURE 3:**
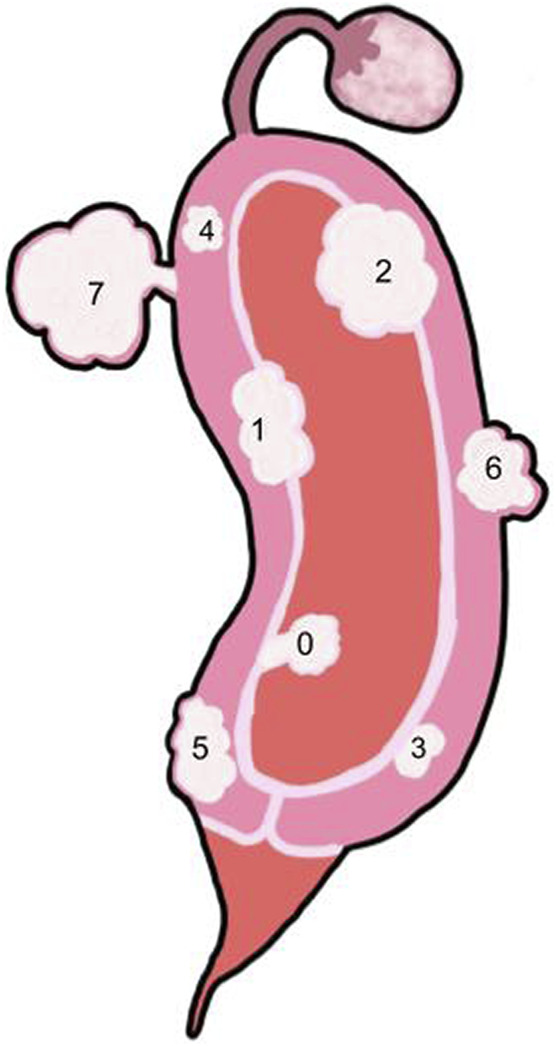
Side view of the uterus with multiple fibroid types -Key: See Table 1. 0: Pedunculated submucosal, 100% of the fibroid is in the uterine cavity. 1: Submucosal, greater than 50% of the fibroid is within the myometrium and the other portion is distorting the endometrium and uterine cavity. 2: Submucosal, less than 50% of the fibroid is within the myometrium and the majority is distorting the endometrium and uterine cavity. 3: Intramural, the fibroid is within the myometrium but touches the endometrium and does not distort the uterine cavity. 4: Intramural, the fibroid is completely with the myometrium. 5: Intramural, the fibroid is predominantly within the myometrium with less than 50% extending outside of the myometrium. 6: Subserosal, greater than 50% of the fibroid is located outside of the myometrium. 7: Pedunculated subserosal, 100% of the fibroid is outside of the myometrium. See Table 1 for classification details.

Fibroids can develop within 3 anatomical parts of the uterus and are classified as subserosal, intramural, and submucosal fibroids (Bajekal, 2000; Cook et al., 2010; Zepiridis et al., 2016) (Figures 1–3). Myomas have been found to originate from the plasticity of myometrial cells during tissue development and maintenance; the cells undergo cellular reprogramming and mutations (Longo and Bulun, 2013; Navarro et al., 2021). According to the International Federation of Gynecology and Obstetrics (FIGO), uterine fibroids are categorized into eight different subtypes (Table 1). The FIGO categorization also has a type 8, which includes lesions on extrauterine locations such as the cervix or broad ligament (Gomez et al., 2021). Subtypes are determined by the position of the myoma in relation to the endometrial cavity.

**TABLE 1:** Classification of uterine fibroids by FIGO.

Classification of uterine fibroids by anatomical positioning		
FIGO	Subtype	Positioning
0	Submucosal - Subtype 0	100% endometrial cavity or intracavity
1	Submucosal - Subtype 1	> 50% intramural
2	Submucosal - Subtype 2	< 50% intramural
3	Intramural	In contact with the endometrium
4	Intramural	100% intramural
5	Intramural	Subserosal >50% intramural
6	Subserosal	Subserosal <50%
7	Subserosal	Pedunculated

### Submucosal

Myomas that cause intramural distortion or reside within the uterine cavity are submucosal fibroids (Puri et al., 2014) (Figures 2, 3). Submucosal fibroids disrupt the endometrial blood supply, which impacts implantation of the embryo (Garcia and Tureck, 1984; Eldar-Geva et al., 1998). In a systematic review completed by Pritts et al., submucosal fibroids were found to lower fertility rates. Submucosal fibroids are also likely to be symptomatic, as they can lead to intermenstrual bleeding and hemorrhage (Divakar, 2008; Wilde and Scott-Barrett, 2009; Bulun, 2013; Puri et al., 2014). Submucosal fibroids can negatively impact the implantation rates of assisted reproductive technology (ART) because the uterine cavity is occupied (Eldar-Geva et al., 1998; Guo and Segars, 2012).

#### Intramural

Intramural fibroids reside in the myometrium cavity without distorting the endometrial cavity (Wilde & Scott-Barrett, 2009) (Figures 2, 3). Intramural myomas impact the establishment of early pregnancy (Eldar-Geva et al., 1998; Pritts et al., 2009). Intramural fibroids produce significantly lower pregnancy rates, implantation rates, and ongoing pregnancy/live birth rates and even significantly higher rates of spontaneous abortion (Pritts et al., 2009). This effect on implantation is seen even when the fibroid does not reach the uterine cavity (Zepiridis et al., 2016; Farhi et al., 1995; Ramzy et al., 1998; Surrey et al., 2001; Jun et al., 2001). One study found that in women who underwent myomectomy, intramural fibroids were the most common type of fibroid to be removed (Casini et al., 2006).

### Subserosal

Subserosal fibroids reside predominantly outside the myometrium (Klatsky et al., 2008) (Figures 2, 3). Subserosal myomas have been found to impact the establishment of early pregnancy (Pritts et al., 2009). However, they have been associated with a very minimal effect on fertility (Zepiridis et al., 2016). Women with subserosal fibroids were found to have no significant differences from those without fibroids (Pritts et al., 2009). Subserosal fibroids tend to be asymptomatic unless they are large, which can cause substantial pressure or pain (Bulun, 2013; Gomez et al., 2021).

### Pedunculated

Fibroids of the final subtype do not reside in a specific location. Pedunculated fibroids can occur both within and outside the uterine cavity (Klatsky et al., 2008), and they are attached to the uterus by a vascular stalk (Gomez et al., 2021) (Figures 2, 3). These fibroids are likely to be asymptomatic unless they are torsioned (Divakar, 2008; Wilde and Scott-Barrett, 2009), but they can also become symptomatic if they grow and begin to push on other masses or detach and become parasitic to the pelvis (Gomez et al., 2021). Parasitic myomas are rare cases where a pedunculated subserosal myoma detaches from the uterus and develops an alternative blood supply from other sources, such as the omental or mesenteric vessels (Cucinella et al., 2011).

### Fibroid cell types and architecture

**FIGURE 4:**
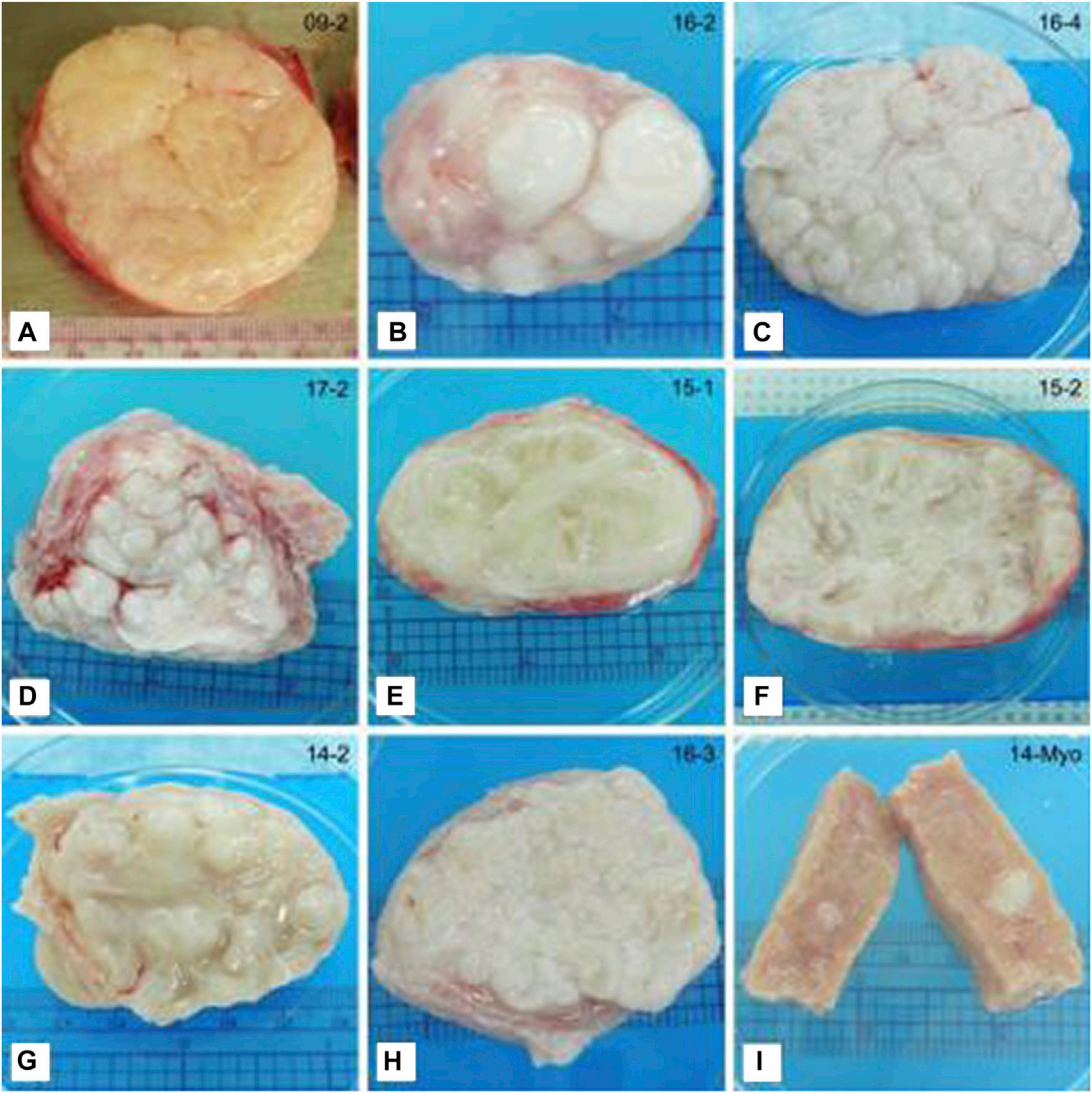
Representative photographs of tissue slices showing differences in the gross appearance of fibroids. **(A)** Classical irregular whorled pattern. **(B–D)** Patterns of nodules. **(E,F)** Trabecular structures. **(G)** Characteristics of multiple patterns. This example shows a trabecular/nodular pattern. **(H)** Not categorized. This example shows a tightly gyrated pattern. **(I)** Myometrial tissue shown for comparison. Note the seedling fibroid embedded in the tissue (white). Ruler (cm) shown for size. This figure and description were adapted from Jayes et al., 2019.

Fibroids have several specific cellular characteristics (Figure 4). A study performed in mice found myometrial proliferation of fusiform smooth muscle cells in the tissue area of the tumor (Romagnolo et al., 1996). The cytoplasm and nuclei of the tumor cells had a normal appearance but displayed high mitotic factors (Romagnolo et al., 1996). There were several fibrous stroma, and within each stroma, spindle cells with high cell proliferation and fibrosis occurred simultaneously. Additionally, it was suggested that fibroids caused narrowing of the lumen in the uterine horn based on their placement (Romagnolo et al., 1996).

Broader slicing of fibroid tissues reveals various patterns in the tissues (Figure 4). These patterns fit into a few main categories. One is the whorled pattern, which is spiraled or twisted; this is the typical pattern expected for fibroids (Khan et al., 2014). In a study that sliced 19 fibroid tissues, approximately 40% of the fibroids examined displayed a whorled pattern (Jayes et al., 2019). Furthermore, approximately 50% displayed a nodular pattern with numerous nodes ranging in size. Another 50% displayed an interweaving trabecular pattern, which is beam-like. Many of the tissues displayed multiple patterns and were classified in both categories (Jayes et al., 2019).

### Effects

Uterine fibroids also play a drastic role in various aspects of reproductive health; they cause approximately 5%–10% of infertility cases (Desai & Patel, 2011; Guo and Segars, 2012; Zepiridis et al., 2016). This can be caused by the location of fibroid growth, which can block the fallopian tubes and disable the passage of the gamete. In addition, fibroids can impact the success rate of women using assisted reproductive technologies (ARTs) (Eldar-Geva et al., 1998; Guo and Segars, 2012).

However, due to the increased hormones during pregnancy, many pregnant women tend to experience large growth of their fibroids. Importantly, after giving birth, 70% of *postpartum* women experience shrinkage of their fibroids (Laughlin et al., 2011; Guo and Segars, 2012; Delli Carpini et al., 2019). This shrinkage is hypothesized to be caused by uterine ischemia when the placenta is torn from the uterine wall, causing immense blood loss. To alleviate this blood loss, the uterus experiences clotting, reducing blood flow and also cutting off the blood supply to the myomas, causing them to shrink (Burbank, 2004).

Symptomatic fibroids are associated with two other classes of symptoms, abnormal uterine bleeding and pelvic pressure and pain (Stewart, 2001; Al-Mahrizi ad Tulandi, 2007; Khan et al., 2014; Whitaker and Critchley, 2016; Giuliani et al., 2020). Abnormal bleeding tends to occur during menstruation and is known as menorrhagia or hypomenorrhea. This bleeding pattern is prolonged and excessively heavy (Gupta et al., 2014; Ghosh et al., 2018; Sohn et al., 2018), causing women in some cases to have to change sanitary products every hour. This symptom is most often seen in submucosal fibroids due to their location, as mentioned previously. Pelvic pressure and pain are caused by enlargement of the uterus. The placement of fibroids can distort the shape of the uterus. Anterior fibroids have been linked to urinary issues and constipation. Rarely, as mentioned above, pedunculated fibroids can cause pain if there is a torsion (Stewart, 2001).

### Prevalence

Studies have shown that the prevalence of uterine fibroids is difficult to determine. As a majority of the cases are asymptomatic, methods of prevalence determination can impact the incidence recorded. A study found that using only clinical diagnosis, the prevalence of fibroids is approximately 33%, but when using ultrasound, it rises to approximately 50% of women, and with histological assessment, the incidence rises to approximately 77% of women (Okolo, 2008).

Uterine fibroids have been noted to be present at a high rate, especially in Black women. A study conducted approximately 30 years ago found that fibroids are 3 to 4 times more likely to occur in Black women than white women (Marshall, 1997). This percentage difference has been determined to be statistically significant; it is approximately 3 times more likely for Black women to develop fibroids than white women even when adjusted for age (Baird et al., 2003). Another study that included multiple races supported the significantly higher prevalence for Black women. They found a prevalence rate of 25.5% in Black women, 7.5% in white women, 5.8% in East Asian women, and 5.5% in South Asian women (Chibber et al., 2016). Studies that include Black, white and Asian women are sparse and generally focus on Black and white women. This may be due to the lower prevalence of fibroids in the Asian race indicated in the literature. Recent studies in the United Kingdom have recorded a rate of 70% of white women and approximately 80% of Black women suffering from uterine fibroids (Bulun, 2013; Khan et al., 2014; Florence and Fatehi, 2022). Furthermore, studies have shown that Black women are more likely to have multiple fibroids. In a study performed in 28 hospitals in Maryland, 57% of Black women had seven or more fibroids, whereas 36% of white women had seven or more fibroids (Kjerulff et al., 1996).

In addition to race being an established risk factor, early age at menarche has been associated with an increased risk of uterine fibroids (Dragomir et al., 2010; D'Aloisio et al., 2010; Faerstein, 2001; Wise, 2004; Marshall et al., 1998; Samadi et al., 1996). However, the cumulative incidence of UFs increases as women approach menopause to more than 80% (Cramer, 1990; Baird et al., 2003). This finding could provide further clinical implications for studies.

### Causes

The causes of leiomyomas are not well known, and research is still needed to understand their formation. However, some drivers of the disease are discussed below.

## Non-hormonal

### Genetic modifications

Several studies point to specific genetic mutations that lead to the development of fibroids, specifically the “MED12, HMGA2, COL4A5/COL4A6, FAS or FH genes” (Eggert et al., 2012; Segars et al., 2014). MED12 is one of the more frequently studied genes. MED12, the gene that codes for the mediator subunit 12 protein, is found on chromosome X. Alterations to MED12 have been found in the majority of women with fibroids in whom chromosomal changes have been noticed (Markowski et al., 2012; Bulun, 2013). These alterations can range from clonal chromosomal abnormalities to simple or complex rearrangements or deletions. In addition to MED12, another common alteration that has been found in fibroids with chromosomal changes is HMGA2. HMGA2 codes for the high mobility group AT2 hook proteins. In some cases of fibroid chromosomal rearrangement, the HMGA2 locus has been targeted and upregulated. MED12 and HMGA2 make up approximately 80%–90% of fibroids with chromosomal abnormalities, but these two alterations are mutually exclusive (Markowski et al., 2012; Bulun, 2013).

Additionally, approximately 40% of women with these tumors have chromosomal abnormalities in “trisomy 12, translocation involving chromosomes (t12; 14) (q14–q15; q23–q24), deletions on chromosome 7 (q22q32), 3q and 1p, and rearrangements of 6p21, 10q22 and 13q21–q22” (Hodges et al., 2002) (Table 2). In a study of Japanese women, there was an association between chromosomes 10, 11, and 22 and leiomyomas, and in white women, there was an association with chromosome 17 (Ordulu, 2016). In a UK-based study, the United Kingdom-based biobank that contains Icelandic (Rafnar et al., 2018) and Finnish (Välimäki et al., 2018) data found a variant among loci in chromosomes 16 and 22 in white women. A study on African women and European women also found an association between chromosome six and fibroids (Giri et al., 2017). In Black women, there was a strong link between chromosomes 22 and eight and fibroids (Hellwege et al., 2017). An additional study by Zhang et al., 2015, found an association between chromosome one and fibroids in Black women.

**TABLE 2:** Chromosomal associations by race.

Race	Chromosomes/Genes	Sources
White	17, 6, 16, 22	Ordulu, (2016) Giri et al. (2017) Rafnar et al. (2018) Välimäki et al. (2018)
Asian	10,11,22	Ordulu, (2016)
Black	6, 22, 8, 1	Giri et al. (2017) Hellwege et al. (2017) Zhang et al. (2015)
Population	MED12, HMGA2, COL4A5/COL4A6, FAS or FH genes	Segars et al. (2014) Eggert et al. (2012) Bulun, (2013) Markowski et al. (2012)

#### Inflammation

Another factor that has been linked to fibroid development is inflammation. Studies on the association between chronic inflammation and leiomyomas are minimal. One study conducted by Protic et al. found an abundance of CD68-positive macrophages, which are associated with inflammation, and inflammatory cells in leiomyoma tissues. They found that there were far more CD68 macrophages in leiomyomas and their surrounding tissues than in the distant myometrium. Furthermore, they found an abundance of inflammatory cells in early-stage cellular leiomyomas, thus forming a link between leiomyomas and inflammation (Protic et al., 2016). Fibrotic disorders such as uterine fibroids are associated with altered ECM pathology, which can be a result of excessive wound healing initiated by the inflammatory response (Zannoti et al., 2021). The results of a study by Kabodmehri et al., 2022, which looked at chronic endometriosis and uterine fibroids, displayed different results than hypothesized. Women with fibroids showed a higher rate of chronic endometriosis than those without fibroids, but that difference was not significant. Within the fibroid group, women with submucosal fibroids were more likely to have endometriosis than women with subserosal or intramural fibroids (64% vs. 37%), and this difference was significant (Kabodmehri et al., 2022). The sample size in the Kabodmehri et al. study was small, but it drew attention to the overall role inflammation plays. If inflammation is frequent in women with submucosal fibroids, then it could play a role in the excessive bleeding that these women experience.

#### Hormonal

Cholesterol-based hormones have been shown to impact tumor growth (Obochi et al., 2009; Chimento et al., 2019). Such hormones include progesterone, estradiol, and vitamin D3 (Table 3). Furthermore, estradiol and progesterone work together to maintain viability for tumor development (Ishikawa et al., 2010; Reis et al., 2016). Progesterone completes the development and proliferation of leiomyomas (Kim et al., 2009; Ishikawa et al., 2010; Reis et al., 2016), and estradiol increases the availability of progesterone receptors on the cells and allows for more sensitivity to progesterone, thus increasing development (Ishikawa et al., 2010; Kim et al., 2013; Reis et al., 2016). These studies do not provide vast racialized data, often only focusing on one or two races.

**TABLE 3:** BMI and hormonal levels by race.

Hormonal levels by race				
	Black	White	Asian	Sources
Average BMI	32.4 $kg/m^{2}$	29.0 $kg/m^{2}$	24.7 $kg/m^{2}$	Liu et al. (2021)
	33.1 $kg/m^{2}$	29.2 $kg/m^{2}$	–	Thomas et al. (2013)
	32.2 $kg/m^{2}$	–	–	Dodgen and Spence-Almaguer, (2017)
	–	–	25.54 $kg/m^{2}$	Zhou et al. (2020)
Estradiol Level	166 pg/ml	142 pg/ml	156 pg/ml	Pinheiro et al. (2005)
	136.1 pg/ml	115.9 pg/ml	–	Haiman et al. (2002)
	225.2 pg/ml	191.5 pg/ml	–	Marsh et al. (2011)
	21.4 pg/ml	–	16.6 pg/ml	Song et al. (2018)
	–	359 pg/ml	547 pg/ml	Huddleston et al. (2011)
	–	–	195.66 pmol/L	Needham et al. (2015)
	–	–	74.1 pmol/L	Ausmanas et al. (2007)
Progesterone Level	1321 ng/d	1289 ng/d	1205 ng/d	Pinheiro et al. (2005)
	15.0 ng/ml	11.0 ng/ml	–	Haiman et al. (2002)
Vitamin D Level (25(OH)D)	20.3 ng/ml	26.7 ng/ml	–	Alzaman et al. (2016)
	18.3 ng/ml	38.0 ng/ml	–	Zhu et al. (2016)
	–	–	19.15 ng/ml	Siddiqee et al. (2021)
	–	–	53.7 nmol/L	Chen et al. (2017)
	–	–	45.1 nmol/L	Wei et al. (2019)

### Estradiol

Cancerous diseases, such as breast cancer, are heavily impacted by hormones such as estradiol and progesterone. Additionally, there are racial differences in hormone levels. Black women had the highest level of estradiol at approximately 166 pg/ml adjusted for BMI, whereas white women had an adjusted level of approximately 142 pg/ml. Asian women were in the middle, with 156 pg/ml (Pinheiro et al., 2005) (Table 3). Several other studies have confirmed this trend. A Haiman et al., 2002, study reported estradiol levels in Black women of 136.1 pg/ml and in white women of 115.9 pg/ml. A study with Asian and white women reported estradiol levels of 547 pg/ml for Asian women and 359 pg/ml for white women, which were again higher for Asian women than for white women (Huddleston et al., 2011). A study with Black and Asian women reported estradiol levels in Black women of 21.4 pg/ml and in Asian women of 16.6 pg/ml (Song et al., 2018). Although these estradiol concentrations were low, they still showed a similar trend as that observed in the Pinheiro study, with Black women having a higher estradiol concentration than Asian women.

### Progesterone

Progesterone is vital to the growth of fibroids, as it works to proliferate cells and maintain their rapid growth (Kim et al., 2009; Ishikawa et al., 2010; Reis et al., 2016). Black women had a concentration of 1,321 ng/d adjusted for BMI, white women had an adjusted concentration of 1,289 ng/d, and Asian women had the lowest concentration of 1,205 ng/d (Pinheiro et al., 2005). Although these differences were not statistically significant, there was a difference (Pinheiro et al., 2005). This kind of difference was also seen in the Haiman et al., 2002, study, where Black women again had the highest progesterone levels of 15.0 ng/ml and white women had a level of 11.0 mg/ml (Table 3).

#### Vitamin D

Another discovered cause of uterine fibroids appears to be a lack of vitamin D. One study found that Black women are severely more likely than white women to be vitamin D deficient, with 42% of Black women being deficient and only 4% of white women being deficient (Kakarala et al., 2007). Using an assay of 25-hydroxyvitamin D (25(OH)D), which is a commonly recognized marker of vitamin D, researchers were able to determine the status of vitamin D in women. The results showed that only 10% of black women and 50% of white women had sufficient vitamin D levels, and women with sufficient vitamin D levels were 32% less likely to have fibroids than women who were deficient (Baird et al., 2013). An optimal level of vitamin D is 25(OH)D at 40–60 ng/ml (Ciebiera et al., 2018).

1,25-Dihydroxyvitamin D3, a biologically active form of vitamin D3, has been shown to decrease tumor proliferation and can induce apoptosis in cancer cells (Halder et al., 2012). Vitamin D is introduced into the body through the skin from UV rays or from 7-dehydrocholesterol or dietary resources in the inactive form. In the liver, it is converted to 25(OH)D and then converted to the active form of 1,25(OH)D in the kidney. It is then carried by vitamin D-binding protein (VDBP) to the skin and different cells (Pike & Christakos, 2017; Ciebiera et al., 2018). VDBP is necessary for maintaining “circulating vitamin D and modulation of the bioavailability, activation, and end-organ responsiveness of the hormone and its metabolites” (Alzaman et al., 2016).

White Americans had a vitamin D level of 26.7 ng/ml in the body, whereas Black Americans had a level of 20.3 ng/ml (Alzaman et al., 2016). Asians may lie in the middle in terms of vitamin D levels, although it is unclear, but they do show a lower level of vitamin D than whites (Siddiqee et al., 2021). However, a population study showed that from 1999 to 2018, white people had a crude cancer rate of 559.1 per 100,000 people, and Black people had a rate of 408.5 per 100,000 people (CDC Wonder). This contradicts the association between VitD levels among the various races, as shown in Table 3.

### Lifestyle

Some studies have associated obesity with uterine fibroids. A literature review performed by Qin et al., 2021, including relevant literature from 1992 to 2020, found a positive correlation between obesity and fibroids. A study on Ghanaian women also showed that there is a greater likelihood of fibroids in obese women (Sarkodie et al., 2016). One study found that obese women accounted for 24% of those undergoing myomectomies and hysterectomies for leiomyomas (Camanni et al., 2010). It is important to note that the correlation has not been completely determined, as some studies have found no association between BMI and fibroids. Possible explanations for obesity increasing the incidence of uterine fibroids include altered sex hormone metabolism, reduced sex hormone binding globulin (SHGB) level, and systematic inflammation (Soave and Marci, 2018).

In terms of altered sex hormone metabolism, it is hypothesized that since adipose tissue is known to affect endocrine tissue, an increase in body fat would increase the amount of estrogen in the body, and estrogen is a driver for uterine fibroids (Soave and Marci, 2018). The reduced SHGB level poses a similar problem, because if there are fewer binding sites, there will be more free estrogen in the bodies of obese women. Finally, systemic inflammation could be a driver, as when fat cells accumulate, there is an increase in inflammatory cytokines in the body that could drive the growth of ECM in fibroids (Soave and Marci, 2018). Various studies show that Black women have the highest average BMI, followed by white women and Asian women, at approximately 32.6, 29.1, and 25.1, respectively (Thomas et al., 2013; Zhou et al., 2020; Liu et al., 2021) (Table 3).

Hormonal levels seemed to fluctuate between the BMI categories, but some overall trends were observed for estradiol and vitamin D (Table 4). In terms of estradiol, some of the findings contradict what is hypothesized about the correlation between estradiol and BMI. In a study by Freeman et al., it was observed that as BMI increases, estradiol level decreases. This refutes the previous hypothesis that estradiol increases as BMI increases. Although a study done by Key et al., 2003, supports the hypothesis of estradiol increasing as BMI increases, they found an estradiol level of 34.8 pmol/L for women with a BMI ≤24 $kg/m^{2}$ **,** 43.2 pmol/L for women with a BMI of $25-29\;kg/m^{2}$ , and 54.9 pmol/L for women with a BMI $\geq 30\;kg/m^{2}$ (Table 4).

**TABLE 4:** Hormonal levels by BMI.

Average BMI				
	≤24 $kg/m^{2}$	$25-29\;kg/m^{2}$	$\geq 30\;kg/m^{2}$	Sources
Estradiol Level	37.7 pg/ml	33.6 pg/ml	30.3 pg/ml	Freeman et al. (2010)
	34.8 pmol/L	43.2 pmol/L	54.9 pmol/L	Key et al. (2003)
	278.9 pg/ml	279.9 pg/ml	258.2 pg/ml	Bellver et al. (2022)
	117.81 pg/ml	149.83 pg/ml	—	Esfahlan et al. (2011)
Progesterone Level	0.19 ng/ml	0.17 ng/ml	0.17 ng/ml	Bellver et al. (2022)
	0.96 ng/ml	1.60 ng/ml	—	Esfahlan et al. (2011)
Vitamin D Level (25(OH)D)	90.4 nmol/L	83.3 nmol/L	77.9 nmol/L	Wei et al. (2019)
	84.9	76.5	73.2 (serum)	Lagunova et al., 2009

There is also an unclear association between progesterone levels and BMI. One study found a decrease in progesterone levels as BMI increased, from 0.19 ng/ml in women with a BMI ≤24 $kg/m^{2}$ to 0.17 ng/ml in women with a BMI $25-29\;kg/m^{2}$ and $\geq 30\;kg/m^{2}$ (Bellver et al., 2022). Another study found that progesterone levels increased as BMI increased, from 0.96 ng/ml in women with a BMI ≤24 $kg/m^{2}$ to 1.60 ng/ml in women with a BMI of $25-29\;kg/m^{2}$ (Esfahlan et al., 2011).

In terms of vitamin D, there was a trend of vitamin D levels decreasing as BMI increased in a Wei et al., 2019, study that reviewed the United Kingdom population. Individuals with a BMI ≤24 $kg/m^{2}$ had an average level of 90.4 nmol/L, individuals with a BMI of $25-29\;kg/m^{2}$ had a level of 83.3 nmol/L, and individuals with a BMI $\geq 30\;kg/m^{2}$ had a level of 77.9 nmol/L.

### Treatments

UFs have been associated with fertility complications, and depending on the location of the fibroids, they can contribute to recurrent pregnancy loss (Freytag et al., 2021). Fibroid categorizations are dependent on the location of the fibroid in the uterus, and treatment is determined with consideration of fertility preservation. Currently, the removal of UFs ranges from invasive (hysterectomy, myomectomy) to minimally invasive (uterine artery embolization, high-frequency magnetic resonance-guided focused ultrasound surgery) to non-invasive pharmaceuticals (Table 5). Pharmaceutical therapies are classified by their mechanism: 1) therapies aimed at controlling the symptoms of UFs, such as progestins, oral contraceptives, and antifibrinolytics, and 2) therapies aimed at reducing the size of fibroids, such as gonadotropin-releasing hormone agonists and antagonists. However, these therapies are not curative (Soliman et al., 2015).

**TABLE 5:** Uterine fibroid treatments.

Treatment type	Procedure type	Description	References/Source
Surgical	Endometrial ablation	Endometrium thickness reduction	Florence and Fatehi, (2022) Giuliani et al. (2020)
	Uterine artery embolization	Blood flow reduction	Florence and Fatehi, (2022) Yerezhepbayeva et al. (2022) Giuliani et al. (2020)
	High-frequency magnetic resonance-guided focused ultrasound surgery	Fibroid size reduction	Yerezhepbayeva et al. (2022) Khan et al. (2014) Keserci et al. (2020) Duc et al. (2018)
	Myomectomy	Fibroid removal	Florence and Fatehi, (2022) De La Cruz & Buchanan, (2017) Giuliani et al. (2020)
	Hysterectomy	Uterus removal	Stewart et al. (2016) Bala et al., 2015 Giuliani et al. (2020)
Hormonal	Mifepristone, Prollex, asoprismil, ulipristal acetate	SPRM	Farris et al. (2019) Donnez et al. (2019) Doherty et al. (2014)
	Combined oral contraceptives	Control menstrual bleeding	Florence and Fatehi, (2022) Kashani et al. (2016) Khan et al. (2014) Giuliani et al. (2020)
	Leuprolide acetate, centrorelix, gamirelix, elagolix, relugolix, linzagolix	GnRH antagonists	Kashani et al. (2016) De La Cruz & Buchanan, (2017) Bulun, (2013) Giuliani et al. (2020) Schlaff et al. (2020) Al-Hendy et al. (2021) Donnez and Donnez (2020)
	Vitamin D	Fibroid size reduction	Baird et al. (2013) Khan et al. (2014)
Non-hormonal	Tranexamic acid	Menstrual bleeding reduction	Kashani et al. (2016) Khan et al. (2014)
	Non-steroidal anti-inflammatory drugs	Menstrual bleeding reduction	Kashani et al. (2016)
	Epigallocatechin gallate	Fibroid size reduction	Grandi et al. (2022) Al-Hendy et al. (2021) Khan et al. (2014)

### Surgical

Treatment for fibroids is often surgical, as it has proven to be the most effective method (Ishikawa et al., 2010; Sheng et al., 2020; Florence and Fatehi, 2022). Surgical options depend on the severity of the case (Table 5). One non-invasive option is endometrial ablation, which removes the thickness of the endometrium but requires the use of permanent contraception post-surgery. Another option is uterine artery embolization, which reduces the blood flow to specific fibroids to alleviate symptoms (Levy, 2008; Khan et al., 2014; Giuliani et al., 2020; Florence and Fatehi, 2022). High-frequency magnetic resonance-guided focused ultrasound surgery is another non-invasive option that destroys the fibroid with high-frequency ultrasound (Levy, 2008; Khan et al., 2014; De La Cruz and Buchanan, 2017; Yerezhepbayeva et al., 2022).

A more invasive option is a myomectomy, which will remove the fibroids themselves, although many women require multiple myomectomies for recurrent fibroids (Levy, 2008; Florence and Fatehi, 2022). The most invasive treatment is a hysterectomy, in which the uterus is removed (Clayton, 2006; Levy, 2008; Stewart et al., 2016; Faustino et al., 2017). Unfortunately, approximately ⅓ of hysterectomies performed are due to uterine fibroids (Stewart et al., 2016). An analysis of the hysterectomy trends in India found that fibroids were the cause of 40% of the hysterectomies performed, followed by chronic cervicitis at 13.6% and dysfunctional uterine bleeding at 12%. The study found that there was a wide array of diseases that could require a hysterectomy, but fibroids were the most prominent (Bala et al., 2015). A full hysterectomy is not always needed; if the risk of bleeding out is low or the fibroids are smaller in size, doctors may opt for more conservative treatment in hopes of saving the uterus. As time progresses, there have been more advances in less invasive techniques to attempt and treat fibroids, with hopes that non-surgical treatments will be effective.

#### Hormonal

Progesterone and estrogen modulators along with other hormonal interventions have shown an ability to slow and reduce fibroid growth (Table 5) (Farris et al., 2019). Progesterone modulators include mifepristone, which is a selective progesterone receptor modulator (SPRM) that works to decrease the size of leiomyomas (Farris et al., 2019; Giuliani et al., 2020). Other utilized forms of SPRMs include but are not limited to Proellex and asoprisnil (Farris et al., 2019). Additionally, combined forms of oral contraceptives to regulate both progesterone and estrogen levels in the body have been shown to treat fibroids (Khan et al., 2014; Florence and Fatehi, 2022). Combined oral contraceptive pills are prescribed to women with or without fibroids to control heavy menstrual bleeding. In women with fibroids, oral contraceptives are not expected to shrink the tumor. They work to suppress endometrial proliferation and thus reduce menstrual bleeding (Kashani et al., 2016).

A common treatment to shrink fibroids involves GnRH agonists. These agonists were accepted by the FDA in 1999 in the form of leuprolide acetate for short-term use prior to surgery. GnRH agonists are the synthetic model of the gonadotropin-releasing hormone GnRH. These agonists have been shown to have greater binding affinity and longer half-lives (Kashani et al., 2016). GnRH agonists work to bind to and downregulate the GnRH receptors. This downregulation decreases the production of follicle-stimulating hormone (FSH) and luteinizing hormone (LH) and leads to a hypoestrogenic state, which causes the tumors to shrink. GnRH agonists have been shown to decrease fibroid size by 30%–65% (Kashani et al., 2016). The hypoestrogenic state caused by agonists is not sustainable, and a study showed that women using agonists needed some add-back therapy to reverse some of the symptoms of the hypoestrogenic state (Kashani et al., 2016).

GnRH antagonists have also been used to treat symptomatic fibroids (Levy, 2008; Khan et al., 2014; Kashani et al., 2016). These antagonists work similarly to agonists, but antagonists have an amino acid substitution from the original GnRH and competes with it for the binding sites (Kashani et al., 2016). Antagonists have been shown to decrease the volume and symptoms of the fibroid. A study showed that when used for 19 days, there was a 41% decrease in the volume of the fibroids. A downside of both antagonists and agonists is that they can cause many adverse side effects because of the hypoestrogenic state and cannot be used for an extended period of time. In the United Kingdom, the injectable GnRH antagonists cetrorelix and ganirelix are rarely used, as they have only been part of observational studies (Kashani et al., 2016). One study with nearly 400 women from across the world determined relugolix to be a GnRH antagonist that is suitable for everyday use and has been proven to reduce menstrual bleeding (Al-Hendy et al., 2021). Another GnRH antagonist that has been shown to decrease heavy menstrual bleeding is elagolix (Schlaff et al., 2020).

Finally, an emerging therapy to treat uterine fibroids is vitamin D. In Eker rats, the active metabolite in vitamin D has been observed to stop the proliferation and production of fibroid cells and their extracellular matrix, thus reducing their volume (Baird et al., 2013; Khan et al., 2014; Sheng et al., 2020).

#### Non-hormonal

One non-hormonal treatment is tranexamic acid, which is a lysine derivative that prevents fibrin degradation and stabilizes clot formation (Table 5) (Florence and Fatehi, 2022). Fibrin is necessary to form clots and stop bleeding (Litvinov and Weisel, 2016). Heavy menstrual bleeding is a prevalent symptom for those suffering from uterine fibroids, which affects the coagulation and homeostatic factors of platelets. Thus, tranexamic acid is used to inhibit the activation of plasminogen to plasmin. This inhibition decreases fibrinolysis, clot breakdowns, menstrual flow and blood loss (Khan et al., 2014; Kashani et al., 2016). Tranexamic acid was FDA approved in 2009 and is given to women both with fibroids and without fibroids to treat heavy menstrual bleeding (Kashani et al., 2016).

Another non-hormonal treatment is non-steroidal anti-inflammatory drugs (NSAIDs), which are used to control uterine bleeding. NSAIDs reduce prostaglandin synthesis by inhibiting the cyclooxygenase enzyme. Endometrial prostaglandin receptors are known to promote the growth of new vasculature in tumors, which can lead to abnormal bleeding. Thus, reducing the synthesis of prostaglandin with NSAIDs reduces the amount of menstrual bleeding (Kashani et al., 2016).

Additionally, the green tea extract epigallocatechin gallate (EGCG) has been shown to decrease the size of uterine fibroids both *in vivo* and *in vitro* (Khan et al., 2014; Grandi et al., 2022). EGCG has been shown to provide anti-inflammatory, antiproliferative, antioxidant and anticancer effects (Al-Hendy et al., 2021). These effects help shrink fibroids. One study found a 17.8% uterine fibroid size reduction (Grandi et al., 2022); another study found a 32% size reduction after four months of use (Al-Hendy et al., 2021).

### African perspective

#### Prevalence across africa

**FIGURE 5:**
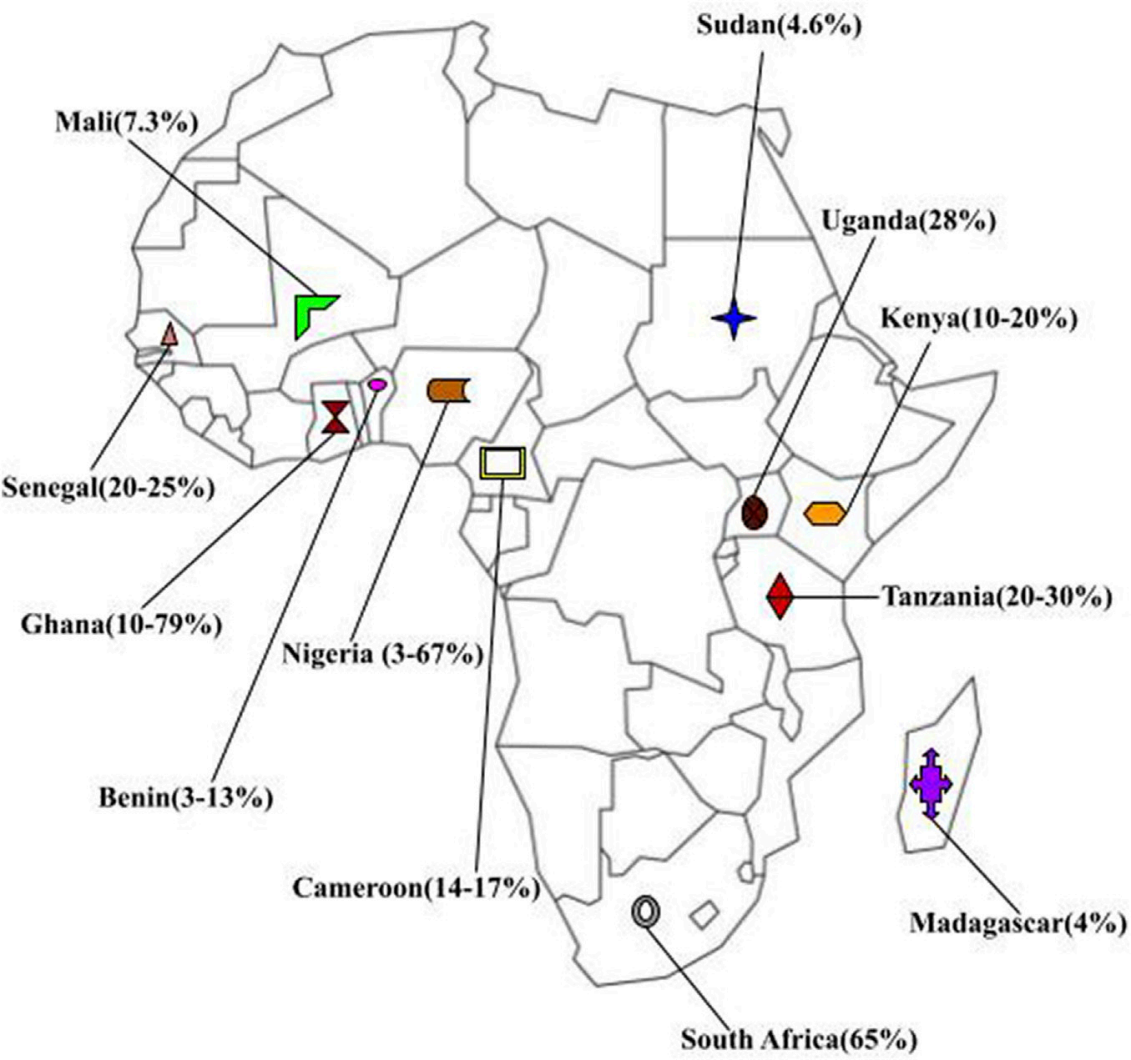
Map of Fibroid prevalence across africa.

On the continent of Africa, other challenges arise for women suffering from uterine fibroids. Upon reviewing several countries on the continent, published documentations of prevalence were difficult to obtain (Figure 5). Notably, there are publications about uterine fibroids by authors across the African continent, but many of them do not include prevalence data (Morhason-Bello and Adebamowo, 2022). This may be due to the lack of medical record digitization until recently.

Furthermore, the rates of uterine fibroid prevalence varied greatly among African countries (Figure 5). Even within countries, the occurrence rates differed vastly. The smallest prevalence was found to be 3.1% in a 5-year study conducted in a teaching hospital in Kano, Nigeria (Muhammad et al., 2013). Over 12,000 women were screened in this study, and 386 of them had leiomyomas. Another study performed in Nigeria with 4500 women found an incidence of 67% (Elugwaraonu et al., 2013). The highest occurrence was found in Ghana, in a study at a teaching hospital in Kumasi where histology showed that 79% of the women had uterine fibroids (Titiloye et al., 2018).

The values found in many countries on the African continent do not match those from the global perspective (Figure 5), particularly those accepted as the prevalence values for Black women. The majority of the studies revealed fibroid occurrences of less than 30% (Mutai et al., 2015; Ernest et al., 2016; Bineta et al., 2018; Hortence et al., 2021; Adawe et al., 2022). There may be severe undercoverage, as some of the available studies do not have access to many regions and are usually conducted in one hospital, resulting in a sampling bias.

### Diagnosis

Typically, to diagnose a fibroid, a woman must come to a hospital or health facility equipped with an ultrasound system (Sarkodie et al., 2016). The combined use of a physical examination and ultrasound helps physicians identify the presence of fibroids (Sarkodie et al., 2016; Igboeli et al., 2019). The determination can also be made using patient history and laboratory investigation. Further testing to detect specific fibroids, such as hysterosalpingography and hysterosonography, can be performed.

There can be challenges in obtaining a diagnosis, some economic and some behavioral. Sociocultural stigma and perceptions and financial handicaps of UF create a barrier to seeking treatment and management of this chronic disease. In a study employed to understand the delay in treatment and diagnosis of disease, many attributed it to perceiving symptoms as “normal” or stating “life must continue” or “bills need to be paid” (Ghant et al., 2016). Furthermore, this delay could be caused by women not having the time, transportation, or money to see a professional (Igboeli et al., 2019). Avoidance-based coping, altered perception of normalcy, limited knowledge of the disease, and lack of financial means deter women from seeking care (Dominic et al., 2019). Thus, women will wait to seek diagnosis until their situation becomes highly symptomatic. Often, they will use orthodox options as a last resort (Okon et al., 2020).

### Treatment

After diagnosis of leiomyomas, treatment is administered. Some women may opt to try traditional practices before an orthodox method (Igboeli et al., 2019; Okon et al., 2020). Orthodox treatments in Africa include “expectant management, surgery, uterine artery embolization, ablative techniques, and medical management” (Akinola et al., 2003; Okon et al., 2020). Of these treatments, surgery is utilized most often. In one study with 656 women seeking gynecological treatment at Korle Bu Teaching Hospital, of those who had fibroids, 79% underwent surgical treatment (Ofori-Dankwa et al., 2019). Furthermore, of the surgical treatments, myomectomy is the most common, with one study reporting that 85% of fibroid treatments were myomectomies and the other 15% were hysterectomies (Okon et al., 2020). Hysterectomy is performed at a lower rate than myomectomy because it eliminates the possibility of further pregnancy. The high usage of surgery as a treatment for uterine fibroids in Africa could be due to numerous reasons. One is that, as discussed previously, women tend to wait to seek a diagnosis, and in those cases, the fibroids tend to be highly symptomatic or large and reduce the possibility of using other treatments. Additionally, as mentioned in the discussion of overall treatments, surgery is the standard of uterine fibroid treatment worldwide, as it has proven to be effective (Florence and Fatehi, 2022).

The non-surgical treatment methods that are being used in other countries, such as progesterone modulators, are too expensive for African women to afford (Igboeli et al., 2019). One study assessed the costs of uterine fibroid treatments in US dollars, and it was found that women who chose surgical options, as opposed to hormonal or non-hormonal options, overall incurred the lowest costs. This was due to fewer missed days of work and less repeated treatment and overall procedure cost (Carls et al., 2008). Additionally, none of these non-surgical treatments offer as permanent an option as myomectomies and hysterectomies (Igboeli et al., 2019). Thus, the women would have to continue ongoing treatment, which could take away time and resources. In terms of treatments such as vitamin D, which can be supplemented, there has not been adequate research to formulate a standard treatment or widespread acceptance of it as a treatment. As research continues, this may become a helpful treatment used worldwide.

## Future directions

Several issues exist surrounding our understanding of and determination of appropriate treatment options for fibroids. First, there is a common understanding that Black women are at a higher risk of developing fibroids than women of any other race. It is therefore necessary to assess the prevalence of the disease on the African continent through a systematic review of medical charts. The data generated can support or refute current understandings and provide options for fibroid prevention and treatment. A medical chart review will provide a clearer picture of the prevailing burden and trend of the disease across generations.

This article suggests that nearly half of uterine fibroids are caused by chromosomal abnormalities. Further research into genetic drivers of the disease, such as chromosomal aberrations, and their stratification by race will shed more light on why the burden is higher for Black women than for white or Asian women. Additionally, by understanding the chromosomal abnormalities that occur, there may be emerging technologies to assess fibroid causes and prevention.

As mentioned previously, the present data covering the association between BMI and fibroids vary. Some studies found no association, while others found significant results showing that fibroids increase as BMI increases. These studies need to be continued with large populations and include data from all races. Such findings are critical now, as average BMI continues to increase across races. Lifestyle is a driver that patients and physicians can actively correct, whether by diet or exercise. A woman with a history of fibroids in her family needs to understand the role that lifestyle can play in the occurrence of the disease. This understanding is especially important for Black women, as they are known to have the highest BMI among other races as well as the highest prevalence of leiomyomas. Incorporating African women into these studies can help determine if the correlation between fibroids and BMI also exists in African women living outside of Western cultures, where their diet and environment are different.

Similarly, a driver that requires further investigation is inflammation. Inflammatory cells are present in the fibroid tissue and extracellular matrix. This may be due to its endogenous origins along the reproductive tract. Effort should be directed at evaluating women with prior inflammatory conditions and assessing whether treating such inflammation can lead to a decrease in fibroid volume or symptoms. Larger population studies will aid in understanding the correlation between fibroids and inflammation and help researchers design better research interventions.

A key research area is understanding the fibroid internal cellular architecture and the various patterns displayed in the tissues. By creating personalized 3D fibroid organelles in the laboratory using tissue samples from women with fibroids, researchers across the African continent can 1) create a fibroid biobank, 2) develop molecular tools to study the drivers of the disease and 3) test both conventional pharmaceutical and herbal drugs. This can help us to study the role of locations in fibroid patterns. Additionally, beyond the patterns identified, these 3D cultures will provide an understanding of the fibroid tissue layers and their ECM. The organelles will continue to produce important biomarkers, which will aid in the establishment of new treatments for the disease and mechanisms to inhibit their proliferation. Furthermore, the establishment of an immortal cell line from African fibroid tissues will be crucial in the sequencing of fibroids and analysis between races. Additionally, by understanding the signaling pathways that fibroid tissue undergoes as it grows in a culture medium, researchers can provide clearer answers to timelines women can expect for their fibroids, especially if specific drivers are more likely to cause fibroids to grow.

Additionally, it has been demonstrated that histological assessments provide the widest scope to accurately identify the prevalence of uterine fibroids. Researchers should aim to develop better diagnostic tools to identify emerging fibroids at an early stage or as the condition changes from asymptomatic to symptomatic.

Non-surgical treatments have proven to be effective in many cases but unfortunately cause severe side effects and often require patients to endure long-term therapy. These treatments tend to be more costly than surgery. Further research should be conducted to help mitigate the side effects of hormonal treatments, provide women with options outside of surgery, and find cost-effective treatment for women. Fibroids have been shown to be highly hormone dependent, which means that women suffering from them could benefit from seeing an endocrinologist. During the annual physical examination, hormone levels should be assessed for high-risk women as a means to identify fibroids, particularly as they evolve from asymptomatic to symptomatic.

The current standard of care, which involves myomectomy and hysterectomy, has evolved with precision medicine. In many advanced countries, myomectomies and hysterectomies can take place laparoscopically, which can lower recovery times and overall costs for women. This is not usually the case in sub-Saharan Africa. As technologies advance, the proper equipment to perform these procedures needs to be expanded worldwide.
